# Buffalo Wins for 1896

**Published:** 1896-01

**Authors:** 


### BUFFALO WINS FOR 1896.

BUFFALO, the Queen City of the Lakes, wins the place of meeting of the United States Veterinary Medical Association for this year, and we are sure that all will now yield, in that the votes have been cast, that this city deserved to be a winner, and we prophesy, early as it may be, that this will prove to be one of the memorable meetings in the history of the national organization. Tapping as a great railroad centre a tier of Northern, Eastern, and Central States, at whose centre, radiating at Buffalo, the Association has never met before, it will prove one of the largest meetings ever held by the parent Association of our land. As a concession to that large number of strong Association men in Northern New York, it will be appreciated with a warmth that will only be equalled by the zealous, untiring efforts of these fellow-colleagues to make this meeting a greater success than any of its predecessors. A beautiful city for a convention in September, one of the greatest live-stock centres in our land, surrounded by some of the most renowned stock-breeding farms in the world; within a few minutes' ride of that great natural wonder, Niagara Falls, with splendid railroad facilities in all directions, all of which will add to the success of the meeting. Best of all, every member of the profession—North, East, South, and West—will receive there the most hospitable welcome, the most zealous attention, and fraternal feeling will be greatly strengthened by the cordiality of the Buffalo veterinarians. But one more point will be added at this time, and that is to say to the members of our organization that it becomes their duty now to prepare plans for being present at the convention of 1896.

